# Perivascular space and white matter hyperintensities in Alzheimer’s disease: associations with disease progression and cognitive function

**DOI:** 10.1186/s13195-025-01707-9

**Published:** 2025-03-18

**Authors:** Philine Marie Schirge, Robert Perneczky, Toshiaki Taoka, Adriana L. Ruiz-Rizzo, Ersin Ersoezlue, Robert Forbrig, Selim Guersel, Carolin Kurz, Matthias Brendel, Julian Hellmann-Regen, Josef Priller, Anja Schneider, Frank Jessen, Emrah Düzel, Katharina Buerger, Stefan Teipel, Christoph Laske, Oliver Peters, Eike Spruth, Klaus Fliessbach, Ayda Rostamzadeh, Wenzel Glanz, Daniel Janowitz, Ingo Kilimann, Sebastian Sodenkamp, Michael Ewers, Boris-Stephan Rauchmann

**Affiliations:** 1 Institute of Neuroradiology, LMU Hospital, LMU Munich, Munich, Germany; 2 Department of Psychiatry and Psychotherapy, LMU Hospital, LMU Munich, Munich, Germany; 3 Sheffield Institute for Translational Neuroscience (SITraN), University of Sheffield, Sheffield, UK; 4 German Center for Neurodegenerative Diseases (DZNE) Munich, Munich, Germany; 5 Department of Neurology, Jena University Hospital, Jena, Germany; 6 Munich Cluster for Systems Neurology (SyNergy), Munich, Germany; 7 Ageing Epidemiology (AGE) Research Unit, School of Public HealthImperial College London, London, UK; 8 Department of Innovative Biomedical Visualization (iBMV), Graduate School of Medicine, Nagoya University, Nagoya, Japan; 9 Department of Nuclear Medicine, Ludwig Maximilian University Hospital, Ludwig Maximilian University of Munich, Munich, Germany; 10 German Center for Neurodegenerative Diseases (DZNE), Berlin, Germany; 11 Department of Psychiatry and Neurosciences, Charité Universitätsmedizin Berlin, Berlin, Germany; 12 ECRC Experimental and Clinical Research Center, Charité– Universitätsmedizin Berlin, Berlin, Germany; 13 Charité– Universitätsmedizin Berlin, Corporate Member of Freie Universität BerlinHumboldt-Universität zu Berlin-Institute of Psychiatry and Psychotherapy, Berlin, Germany; 14 Department of Psychiatry and Psychotherapy, Charité, Charitéplatz 1, 10117 Berlin, Germany; 15 Department of Psychiatry, School of Medicine, Technical University of Munich, Munich, Germany; 16 University of Edinburgh, UK DRI, Edinburgh, UK; 17 German Center for Neurodegenerative Diseases (DZNE)Venusberg-Campus, Bonn, Germany; 18 Department of Old Age Psychiatry and Cognitive Disorders, University Hospital Bonn, Bonn, Germany; 19 Department of Psychiatry, University of Cologne, Medical Faculty, Kerpener Strasse 62, 50924 Cologne, Germany; 20 Excellence Cluster on Cellular Stress Responses in Aging-Associated Diseases, University of Cologne, Cologne, Germany; 21 German Center for Neurodegenerative Diseases (DZNE), Magdeburg, Germany; 22 Institute of Cognitive Neurology and Dementia Research (IKND), Otto-von-Guericke University, Magdeburg, Germany; 23 Institute for Stroke and Dementia Research (ISD), Ludwig Maximilian University Hospital, Ludwig Maximilian University, Munich, Germany; 24 German Center for Neurodegenerative Diseases (DZNE), Rostock, Germany; 25 Department of Psychosomatic Medicine, Rostock University Medical Center, Gehlsheimer Str. 20, Rostock, Germany; 26 German Center for Neurodegenerative Diseases (DZNE), Tübingen, Germany; 27 Department of Psychiatry and Psychotherapy, Section for Dementia Research, Hertie Institute for Clinical Brain Research and University of Tübingen, Tübingen, Germany; 28 Department of Psychiatry and Psychotherapy, University of Tübingen, Tübingen, Germany; 29 Institute of Neuroradiology, University Hospital, LMU Munich, Marchioninistraße 15, 81377 München, Germany

**Keywords:** Perivascular space, Diffusion tensor imaging, Amyloid-beta, Cognitive decline, Alzheimer's disease, Dementia

## Abstract

**Background:**

Alzheimer’s disease (AD) is the leading cause of dementia, characterized by the accumulation of amyloid-beta (Aβ) and neurofibrillary tangles. Recent studies emphasize the role of vascular factors, including the glymphatic system, in AD pathogenesis, particularly in Aβ clearance. The diffusion tensor image analysis along the perivascular space (DTI-ALPS; ALPS-Index) has emerged as a novel, non-invasive method to evaluate the glymphatic system in vivo, showing glymphatic insufficiency in AD. This study aimed to investigate alterations in the function of the glymphatic system in individuals with AD versus healthy controls (HC), and to explore its association with Aβ, cerebrovascular disease (CVD), white matter hyperintensities (WMH), and cognitive function.

**Methods:**

DTI MRI data from three independent study cohorts (ActiGliA: AD *n* = 16, Controls *n* = 18; DELCODE: AD *n* = 54, Controls *n* = 67; ADNI: AD *n* = 43, Controls *n* = 49) were used to evaluate the perivascular space (PVS) integrity; a potential biomarker for glymphatic activity. The DTI-Along the Perivascular Space technique was used to measure water diffusion along PVS providing an index to assess the efficiency of the glymphatic system’s waste clearance function. WMH load was quantified in FLAIR MRI using the lesion segmentation tool. We quantified WMHs volume within our defined region of interest (ROI) and excluded participants with any WMHs to avoid confounding the ALPS-Index. Associations with cerebrospinal fluid (CSF) AD hallmark biomarkers, cognitive performance (MMSE) and clinical severity (CDR) were assessed.

**Results:**

AD patients had a significantly lower ALPS-Index vs. healthy controls (ActiGliA: AD: mean = 1.22, SD = 0.12; Controls: mean = 1.36, SD = 0.14, *p* = 0.004; DELCODE: AD: mean = 1.26, SD = 0.18; Controls: mean = 1.34, SD = 0.2, *p* = 0.035; ADNI: AD: mean = 1.08, SD = 0.24; Controls: mean = 1.19, SD = 0.13, *p* = 0.008). The ALPS-Index was associated with CSF Aβ concentration, WMH number and MMSE and CDR. WMH, found in the ROIs correlated negatively with the ALPS-Index.

**Conclusions:**

This study highlights the potential of the DTI-ALPS-Index as a biomarker for glymphatic dysfunction in AD. It underscores the importance of considering vascular factors and the glymphatic system in the pathogenesis and diagnosis of AD as WMHs in the ROI could cause disturbances and inaccurate indices.

**Supplementary Information:**

The online version contains supplementary material available at 10.1186/s13195-025-01707-9.

## Introduction

Alzheimer’s disease (AD) is a chronic neurodegenerative disease, characterized by the progressive deterioration of neural circuits leading to cognitive decline and dementia. While extensive research on the neuropathological hallmarks of AD, such as amyloid-β (Aβ) plaques, neurofibrillary tangles, and glial responses has been carried out, the importance of vascular contributions has long been recognized but sometimes overlooked in earlier studies [1]. According to the amyloid hypothesis, an imbalance between the production and clearance of Aβ results in the accumulation and aggregation within the brain and triggers a cascade of pathophysiological events with the formation of neurofibrillary tau tangles, loss of neurons and synaptic dysfunction ultimately resulting in dementia [2]. The production of Aβ has received much attention as a therapeutic target against AD in recent years; less emphasis was placed on the modification of mechanisms related to Aβ clearance. It has been postulated that insufficient cerebral clearance of Aβ peptides which are produced at a normal rate account for most cases of sporadic AD [3]. Until recently, transport across the blood-brain barrier (BBB) was considered as the main mechanism to remove Aβ from the brain; however, newer findings support the existence of additional important mechanisms [4]. Bulk-flow of interstitial fluid (ISF) mediated by astroglia and the recently discovered glymphatic vessels in the meninges probably also make a meaningful contribution to Aβ clearance [5]. Previously, it was thought that about 75% of Aβ is cleared by BBB transport and only 10% by the glymphatic system. However, recent photon imaging studies in mice, using microscopy with fluorescent tracers, have suggested that the glymphatic system contributes to a larger part of Aβ clearance than previously thought [6]. The glymphatic system comprises the transport of cerebrospinal fluid (CSF) after para-arterial influx and transport via aquaporin 4 channels into the interstitium, followed by convective intestinal transport to efflux via the perivenous space into the lymphatic system [7, 8]. This system regulated by astrocytes is assumed to play a major role in the drainage of brain metabolites, and its malfunction may lead to the accumulation of waste products and is related to AD pathogenesis [7, 9]. The glymphatic pathway has been visualized in rodents using a variety of imaging approaches; clinical imaging of the glymphatic pathway is an emerging field, and several innovative methods in humans intrathecal contrast agent injection has been used to visualize the glymphatic system [10], a rather invasive imaging approach. Recently a novel technique “diffusion tensor image analysis along the perivascular space (DTI-ALPS; ALPS_Index) using non-invasive MR diffusion tensor imaging (DTI) based technique has been developed to evaluate the glymphatic system in vivo [11]. Numerous studies have shown glymphatic insufficiency in a variety of diseases using this approach, which measures the ratio of water diffusivity along the perivascular space [12–14]. In AD and older adults at risk for dementia a decreased ALPS-Index compared to healthy controls was reported in two previous studies [11, 15].

Besides the dysfunction of the glymphatic system there is increasing evidence suggesting that cerebrovascular disease (CVD) and AD not only share common risk factors, but also have additive harmful effects on cognitive function [16]. Studies have demonstrated that reducing cardiovascular risk protects against AD [17]. A two-hits-mechanism where BBB disruption as a “first hit” is followed by Aβ accumulation as a “second hit” ultimately leading to full-blown AD has been proposed [18]. A vast number of studies demonstrated links between established AD biomarkers and vascular abnormalities in AD. It has been shown that Aβ [19], tau [20] and glucose metabolism [21] in AD are associated with microvascular damage, and it was suggested that vascular changes are among the earlies events in the course of the disease [22]. A frequent finding in AD are white matter hyperintensities (WMH) observable on T2-weighted Fluid-attenuated inversion recovery (FLAIR) brain MRI scans. The pathophysiological origin of WMH has not yet been fully understood. Associations with cerebrovascular damage, microglial and endothelial cell activation and glial reorganization have been reported frequently, suggesting that WMH is a proxy measure of white matter damage [23]. Recent studies have also reported associations between white matter changes and both Aβ [24] and tau pathology [25]. Additionally, the extent of WMH is correlated with cognitive impairment and an increased risk of dementia [26].

In our study, we first examined alterations in the glymphatic system integrity and function, specifically focusing on changes detected using the DTI-ALPS method, across three independent cohorts of individuals with AD compared to healthy controls. Additionally, we investigated the potential contamination of DTI-ALPS measurements by the concurrent presence of WMH in the regions analyzed, often ignored by previous analyses. Previous studies have demonstrated a correlation between the ALPS-Index and both the Mini-Mental State Examination (MMSE) scores, and Aβ as measured in PET imaging [27, 28]. However, to our knowledge, this is the first investigation that explicitly examines how impairments in the glymphatic system is associated with CSF Aβ levels. Furthermore, we assess the associations between glymphatic system impairments and cerebral small vessel disease, evidenced by WMH in FLAIR MRI.

## Methods

### Participants

**Fig. 1:**
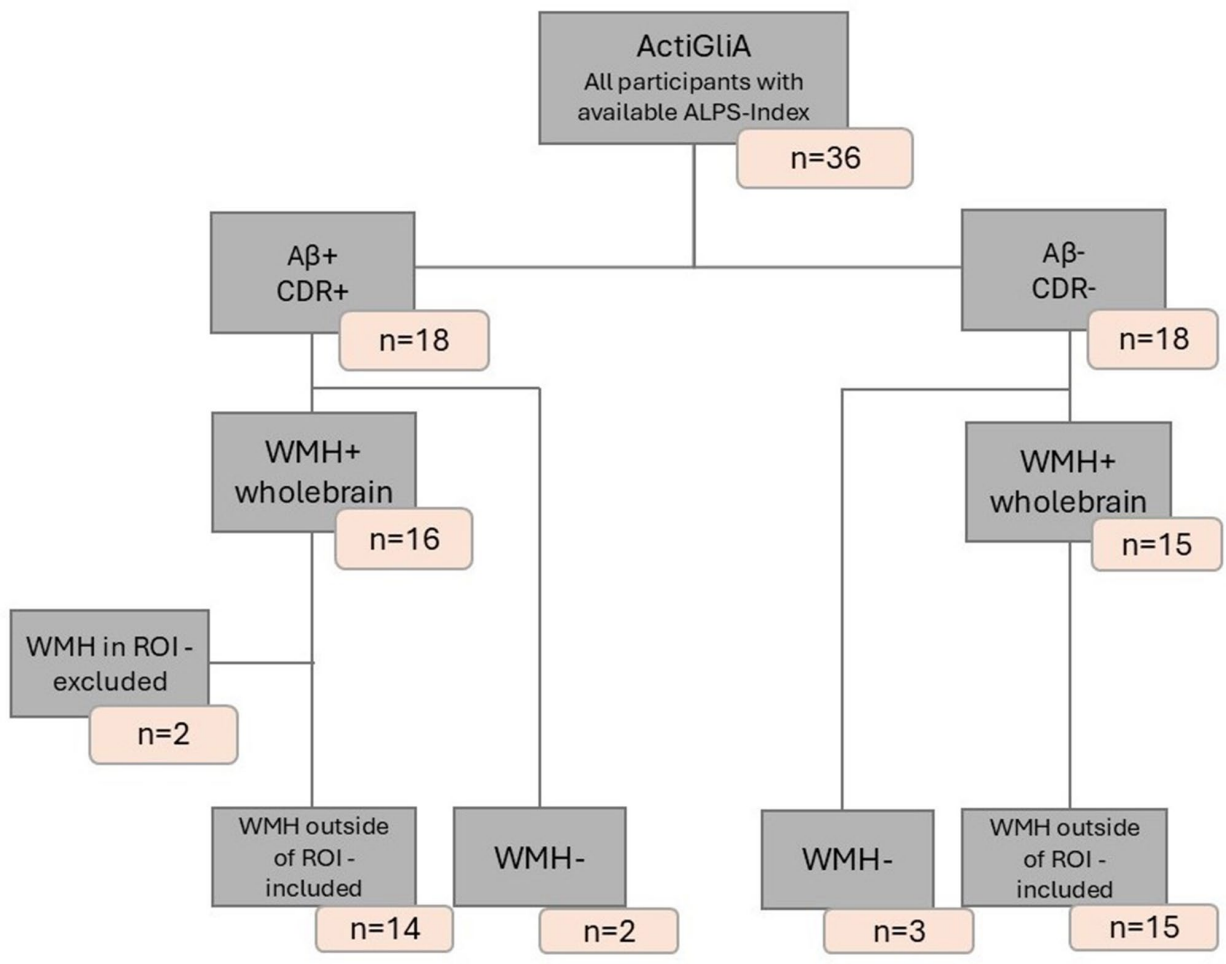
Flowchart of ActiGliA Participant Inclusion and Exclusion Criteria. AD: Aβ+, CDR+; HC: Aβ-,CDR-

**Fig. 2:**
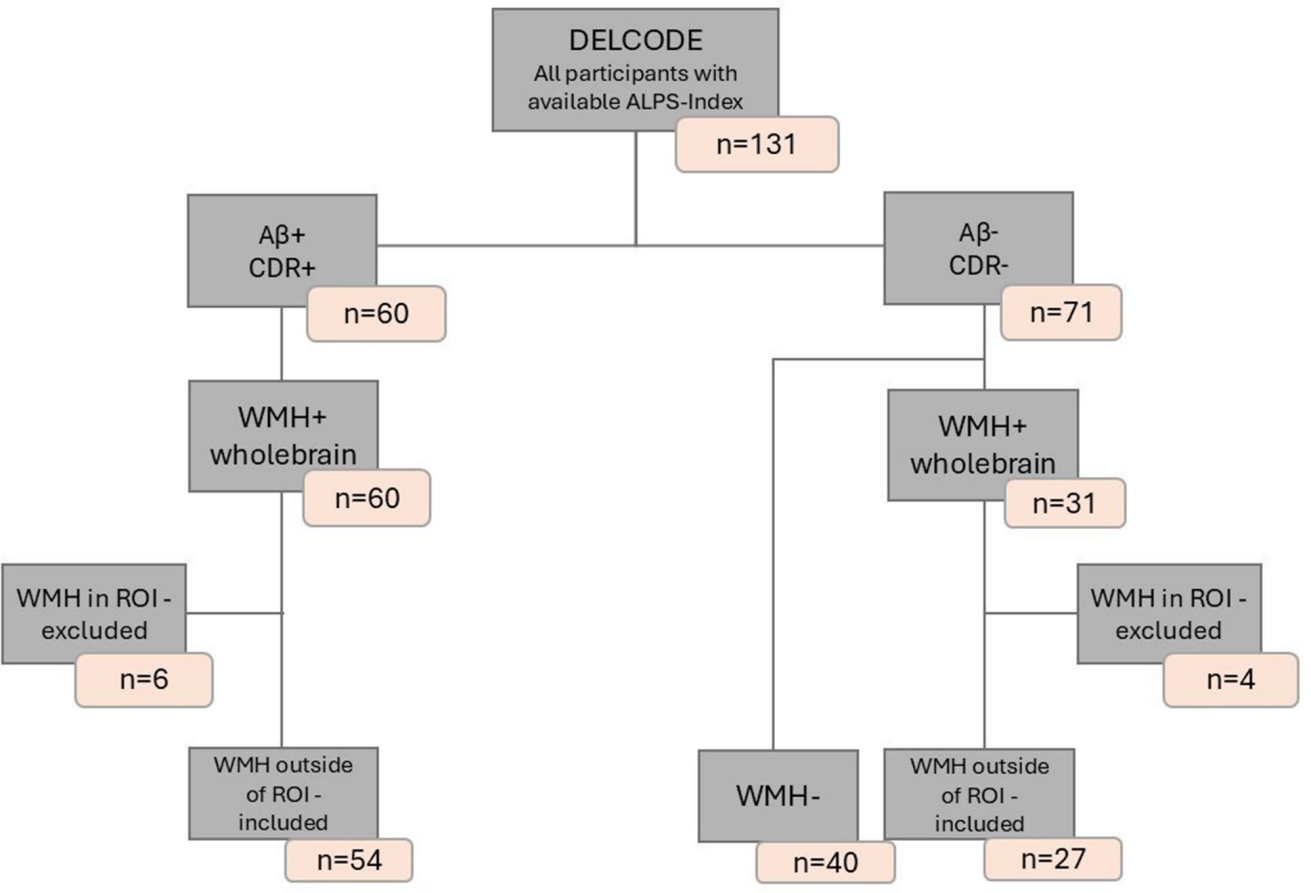
Flowchart of DELCODE Participant Inclusion and Exclusion Criteria. AD: Aβ+, CDR+; HC: Aβ-,CDR-

**Fig. 3:**
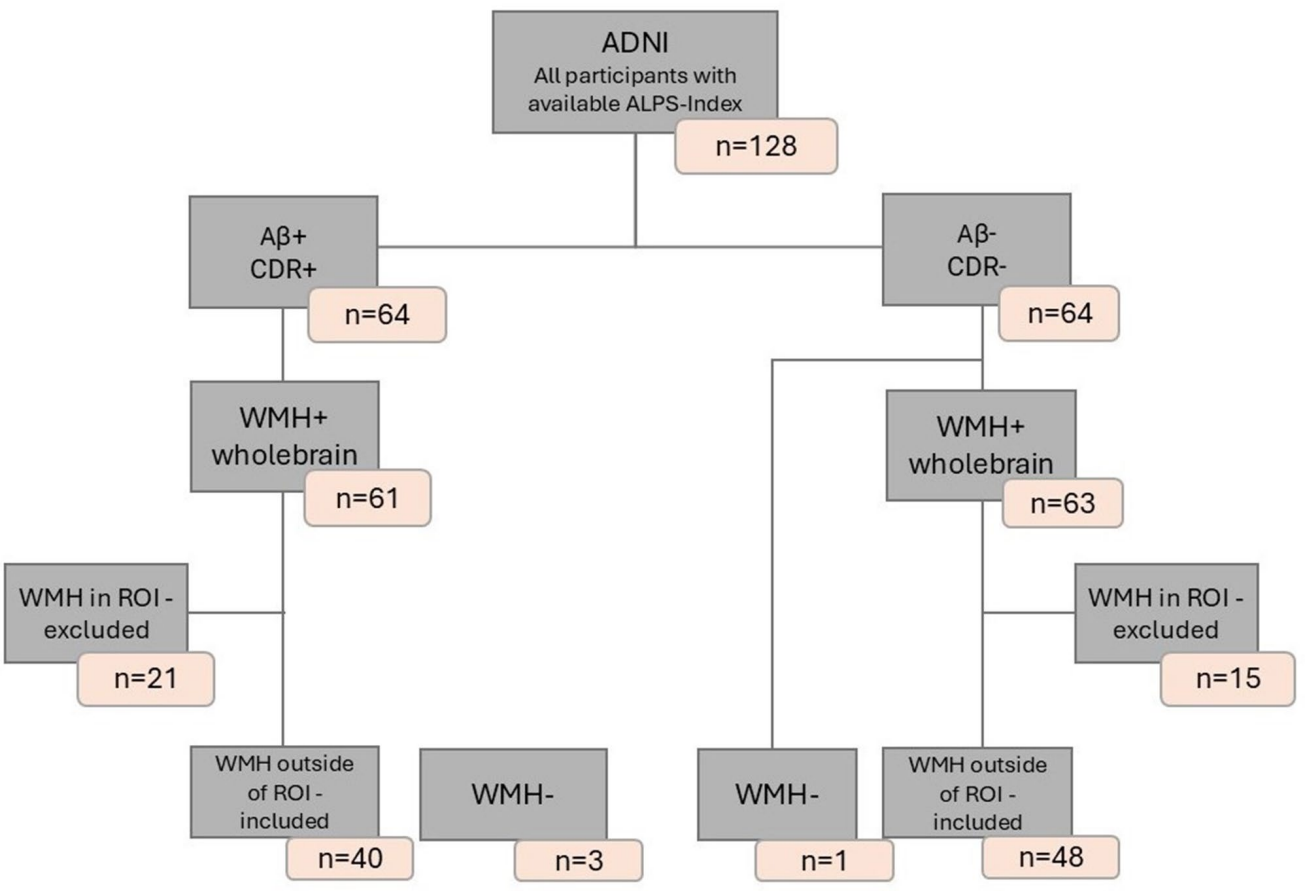
Flowchart of ADNI Participant Inclusion and Exclusion Criteria. AD: Aβ+, CDR+; HC: Aβ-,CDR-

We tested our hypothesis in tree independent cohorts. Participants in all three cohorts were stratified by the following scheme: Alzheimer’s Disease (AD) patients, were defined as participants with a clinical dementia rating (CDR) > = 0.5 [29, 30] and positive Aβ42/40 status, determined by CSF. Healthy controls (HC) were defined as CDR = 0 and Aβ negative for CSF. For the cohort-specific CSF cutoff values, please see the CSF biomarker section. Data from three independent cohorts were analyzed equally to investigate the relationship between glymphatic system function and Alzheimer’s disease. The Activity of Cerebral Networks, Amyloid and Microglia in Aging (ActiGliA) study, a prospective, observational, single-center study of the Munich Cluster for Systems Neurology (SyNergy) at Ludwig-Maximilians-University (LMU) Munich, included *n* = 16 AD patients and *n* = 18 HC [31], as seen in Fig. 1. The German Center for Neurodegenerative Diseases Longitudinal Cognitive Impairment and Dementia (DELCODE) [32] study contributed *n* = 54 AD patients and *n* = 67 HC subjects (see Fig. 2), while the Alzheimer’s Disease Neuroimaging Initiative (ADNI) provided data from *n* = 43 AD patients and *n* = 49 HC, see Fig. 3. Participants from all three cohorts underwent DTI and FLAIR imaging for analysis. Each study was approved by the local ethics committee of the participating centers, including the ethics committee of LMU Munich (project numbers 17–755 and 17–569). Patients with early AD (subjective cognitive impairment, MCI and mild AD dementia) and age-matched cognitively normal controls were included after providing written informed consent in line with the declaration of Helsinki.

### CSF biomarkers analysis

CSF biomarkers were evaluated using established commercially available assay kits, in all three cohorts, using aliquoted samples and a single lot of each reagent for each of the measured biomarkers. The electrochemiluminescence immunoassay (ECLIA) Elecsys cobas e 601 instrument (Roche Diagnostics GmbH, Penzberg, Germany) was used to quantify the CSF concentrations of Aβ42, p-tau181 in the ADNI cohort. CSF peptide measures in the DELCODE and ActiGliA cohort were performed using aliquoted samples using commercially available (Fujirebio, Malvern, PA) enzyme-linked immunosorbent assays (ELISAs). The following cutoffs for CSF Aβ42/40 were applied to determine whether a study participant was amyloid positive: Aβ42/40: <= 0.08 [33] for DELCODE and Aβ42/40: <= 0.05 (ADNI and ActiGliA respectively) [34].

### Clinical and cognitive testing

To evaluate cognitive performance the MMSE was performed [35]. Analysis details are presented in the supplemental material. Clinical dementia severity was determined using the Clinical Dementia Rating (CDR) global score [30].

### MRI acquisition

ActiGliA: MRI data for the entire ActiGliA cohort was acquired at the Department of Radiology of LMU Munich on a Siemens 3T Magnetom Skyra MR system (Siemens Healthineers, Erlangen, Germany). A 0.8 mm isovoxel high resolution T1-weighted structural MRI sequence (repetition time (TR), 2060 ms; echo time (TE), 2.17 ms; flip angle (FA), 12°; field of view (FoV), 240 mm) and a diffusion weighted imaging (DWI) MRI sequence with a multi-band acceleration factor 3 (TR, 3800 ms; TE, 104.8 ms; b-value, 2000 s/mm²; 108 diffusion directions; FA, 90°; FoV, 240 mm) were acquired. Matrix size: 120 × 120 with a voxel size of 2.0 × 2.0 × 2.0 mm and a FoV of 240 × 240 mm.

DELCODE: DWI data were acquired with single-shot echo-planar imaging (EPI) on 3-Tesla MRI scanners (i.e., Siemens MAGNETOM TrioTim, Verio, Skyra, and Prisma; Siemens Healthcare, Erlangen, Germany). Acquisition parameters were the same across all scanners: GRAPPA acceleration factor 2; TR = 12,100 ms; TE = 88.0 ms; b-values = 700 and 1000 s/mm2 (30 directions each); 70 diffusion directions including 10 b = 0 images; FA, 90°; FoV, 240 × 240 × 144 mm3; phase encoding = anterior-to-posterior; matrix size = 120 × 120; voxel size, 2.0 mm isotropic; 72 axial slices; total acquisition time = 14 min 45 s. For the 3D FLAIR sequence, the acquisition parameters were as follows: TR = 5 s, TE = 394 ms, inversion time (TI) = 1.8 s, resolution = 1 × 1 × 1 mm3, matrix size = 256 × 256, 192 slices [36].

ADNI: The ADNI MRI acquisition protocol is reported elsewhere (JG_ADNI3_AAIC_poster_FINAL.pptx (usc.edu). In short, diffusion data were acquired using DWI, on 3-Tesla MRI scanners, following ADNI-3 Basic Protocol, with a geometry of FoV at reconstructed resolution in mm: 232 × 232 × 160; phase encoding = posterior-to-anterior; voxel size, 2 × 2 × 2 mm3; 80 axial slices. Following timing parameters in ms: TE = 56, TR = 7200, and a run time of 7:30 min. Single b-value = 1000 s/mm2, shell b = 0 images interleaved throughout if possible in product sequence. 3D FLAIR was acquired with a geometry of FoV at reconstructed resolution in mm: 256 × 256 × 160, voxel size, 1 × 1 × 1.2 mm3. Timing parameters in ms: TE = 119, TR = 4800, TI = 1650. With an approximate run time in minutes: 5:30. TE definition varies by vendor, effective TE is quoted.

### Preprocessing of the diffusion-weighted imaging and calculation of the ALPS-Index

The ALPS-Index was estimated as previously described [11, 36]. In short, we preprocessed the images by generating diffusivity maps from DTI data in the direction of the x-axis (right-left), y-axis (anterior-posterior), z-axis (inferior-superior), and color-coded fractional anisotropy (FA) maps of each subject. These images were used for the calculation of the ALPS-Index. Four regions-of-Interest (ROIs) with a radius of five-millimeter were then drawn manually in the centrum semiovale along the projection and association fibers orthogonal to the perivascular spaces at the level of the medullary veins on each side of the lateral ventricle on the color-coded FA map. Diffusivity was calculated in the direction of the x-axis, y-axis, and z-axis of the ROIs along the projection fibers and the association fibers as X-proj, Y-proj, Z-proj, X-assoc, Y-assoc, Z-assoc, respectively. The ratio between the mean measures of diffusion (ALPS-Index) were calculated as

$$\:DTIALPS\_index=\frac{({meanX}_{proj}+{meanX}_{asoc})}{({meanY}_{proj}+mean{Z}_{assoc})}$$

We calculated the ALPS-Index on the right and left side separately (ALPS-Index_R/ALPS-Index_L). Diffusivity along the X-axis in these areas have been proposed to represent the perivascular spaces’ diffusivity [11, 36].

### Estimation of white matter lesions

We assessed WMH using MRI FLAIR images. The lesions were segmented using the lesion prediction algorithm [37] implemented in the LST toolbox version*3.0.0* (www.statistical-modelling.de/lst.html) for SPM12. Parameters of this model fit were used to segment lesions by providing an estimate for the lesion probability for each voxel [38].

**Fig. 4:**
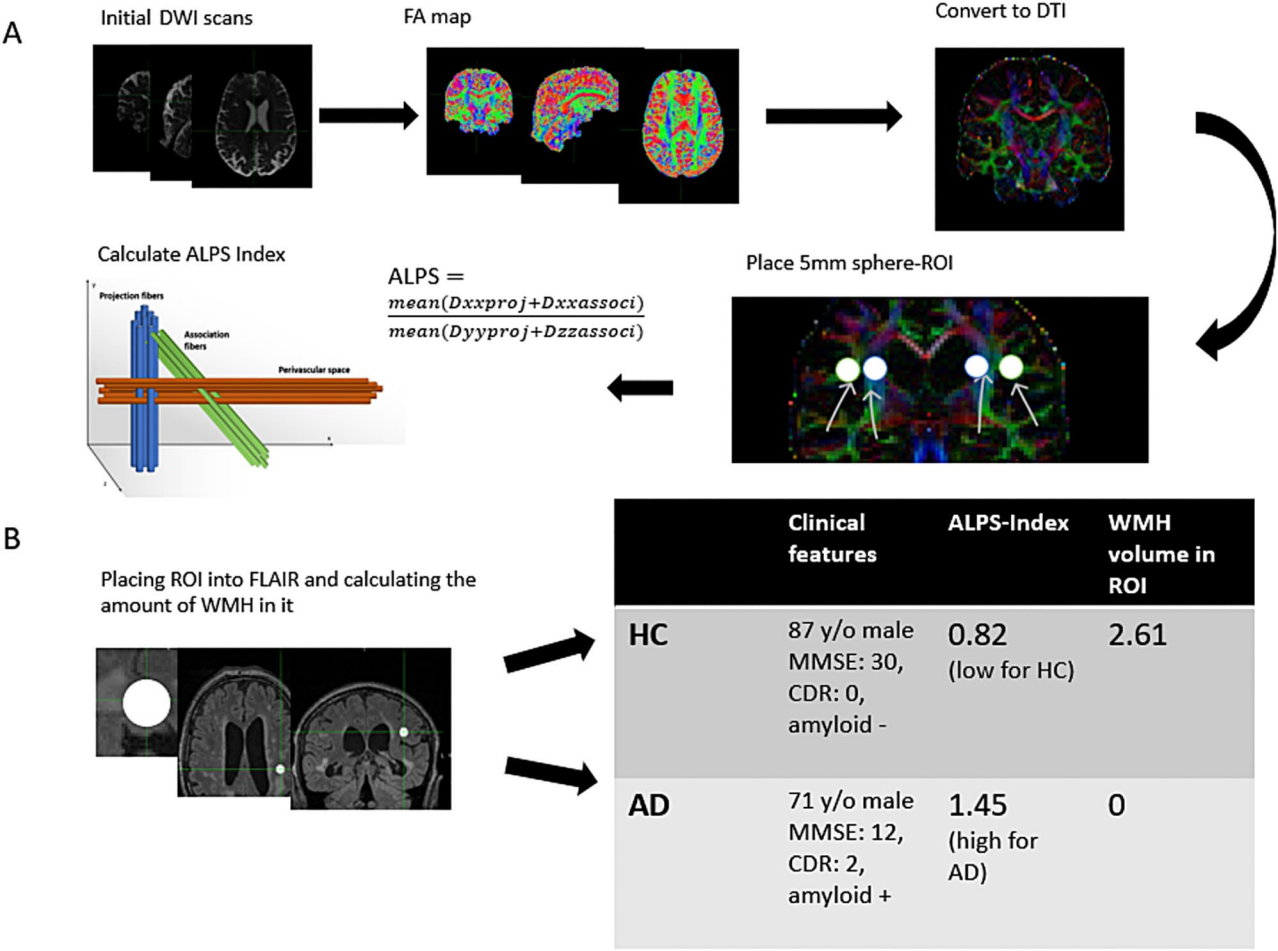
Schematic illustration of the analysis process and data from representative participants. (**A**) Analysis process from initial diffusion weighted imaging (DWI) images to calculate ALPS indices. (**B**) Analysis process to calculate volume of white-matter hyperintensities (WMH) in regions of interest (ROI) and data from 2 representative subjects from ADNI cohort, which illustrates how higher WMH-Volume in ROI might interfere with ALPS-Index in healthy controls. ALPS: diffusion tensor image analysis along the perivascular space

To enhance the robustness of our analysis, we developed a computational script to quantify the presence of WMHs within our defined ROI. In short, this script automates the quantification of WMH in specified ROIs by transforming hand-drawn DTI coordinates into FLAIR space, creating 5 mm spherical masks around these coordinates, and counting the intersecting WMH voxels. It efficiently processes multiple cases, facilitating the analysis of WMH load in defined brain regions. To and mitigate confounding of the ALPS-Index by WMHs, we decided to exclude participants exhibiting any level of WMH in our ROI (*n* = 48) from analyses investigating correlations and associations between the ALPS-Index and biomarkers, WMH volume in the rest of the brain (outside of the ROI), and clinical markers. However, these excluded participants were analyzed separately to evaluate the specific impact of WMH within the ROI on the ALPS-Index. This decision was informed by the observed negative correlations between WMH and ALPS-Index in ADNI and DELCODE, both cohorts with the most WMH detected, which could potentially obscure the interpretation of our findings. For a better understanding of how the images were processed, see Fig. 4. Specifically, we excluded *n* = 36 subjects, including AD and HC, from the ADNI, *n* = 10 from the DELCODE, and *n* = 2 participants from the ActiGliA dataset (see Tables 1 and 2 in RESULTS). The rationale for this exclusion criterion is grounded in the premise that vascular anomalies could erroneously affect the interpretation of ALPS-Index measurements, thereby skewing the assessment of glymphatic function.

**Table 1:** Correlation between ALPS indices and amyloid beta42/40 ratio

	ActiGlia				ADNI				DELCODE			
	Aβ42/40				Aβ42/40				Aβ42/40			
	β	SE	Adj-*R*²	*p*-value	β	SE	Adj-*R*²	*p*-value	β	SE	Adj-*R*²	*p*-value
ALPS_comb	-0.14	0.34	0.31	0.675	-0.17	0.40	0.05	0.670	-0.65	0.24	0.15	0.009*
ALPS_R	-0.08	0.31	0.39	0.796	-0.06	0.35	0.25	0.877	-0.58	0.25	0.11	0.020*
ALPS_L	-0.18	0.35	0.23	0.621	-0.06	0.40	0.06	0.891	-0.56	0.24	0.13	0.022*

*Abbreviations*: ALPS_R, ALPS_L: calculated ALPS index on right R and left L hemisphere of the brain. ALPS_comb: combined ALPS index, by calculating the mean of Index for right and left hemishphere; adj-R²: adjusted R²; SE: Standard error; * significant difference with *P* < 0.05. Calculated with multivariate linear regression analyses, using age, sex, years of education and diagnose (ADvsHC) as confounding variables

**Table 2:** Correlation between ALPS indices and amyloid beta42/40 ratio in AD group

	ActiGlia				ADNI				DELCODE			
	Aβ42/40				Aβ42/40				Aβ42/40			
	β	SE	Adj-*R*²	*p*-value	β	SE	Adj-*R*²	*p*-value	β	SE	Adj-*R*²	*p*-value
ALPS_comb	-0.33	0.03	0.48	0.138	-0.26	8.50	-0.08	0.255	-0.40	2.30	0.23	0.003*
ALPS_R	-0.18	0.04	0.57	0.345	-0.30	5.22	0.07	0.167	-0.45	2.50	0.32	0.000*
ALPS_L	-0.45	0.03	0.10	0.125	-0.13	5.43	-0.06	0.575	-0.26	2.60	0.08	0.062

*Abbreviations*: ALPS_R, ALPS_L: calculated ALPS index on right R and left L hemisphere of the brain. ALPS_comb: combined ALPS index, by calculating the mean of Index for right and left hemishphere; adj-R²: adjusted R²; SE: Standard error; * significant difference with *P* < 0.05. Calculated with multivariate linear regression analyses, using age, sex, and years of education as confounding variables

### Statistical analysis

Group differences were analyzed using an analysis of covariance (ANCOVA) adjusted for age and sex. Statistical analysis was performed using Python 3, including the libraries NumPy, Pandas, and Matplotlib. Associations between ALPS-Index and Aβ, WMH, CDR and MMSE were analyzed using multiple linear regression models in SPSS version 27 using age, sex and years of education and diagnose (AD vs. HC) as confounding variables. Statistical significance was considered at an alpha level = 0.05. To address the non-normal distribution of WMH volume data, we applied a log-transformation before conducting t-tests or multiple linear regression analyses.

## Results

**Fig. 5:**
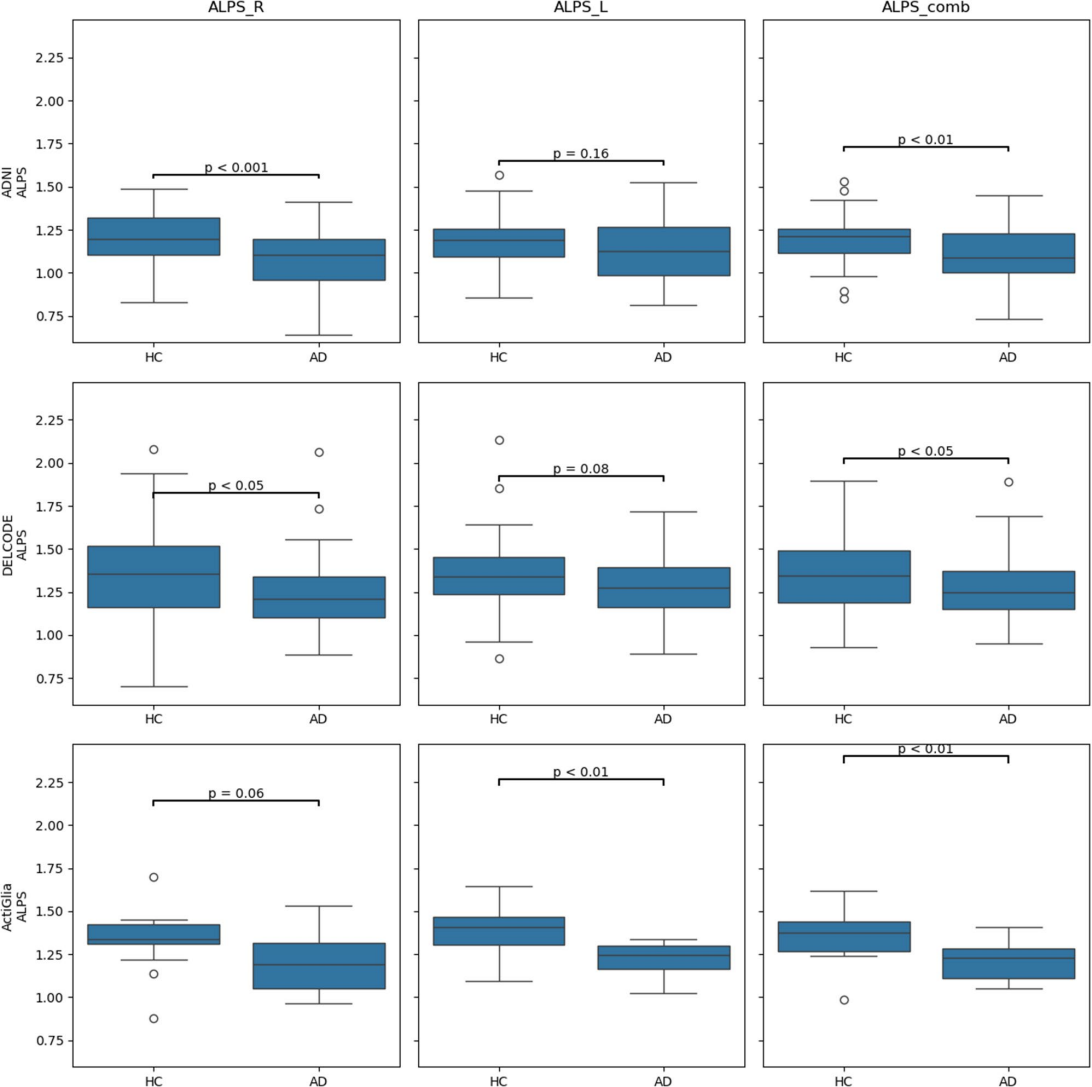
Boxplots in all 3 cohorts comparing ALPS-Indices combined, left side and right side amongst patients with Alzheimer’s dementia (AD) and healthy control participants (HC)

The sociodemographic and clinical characteristics of the three cohorts (ActiGliA, DELCODE, ADNI) are presented in Supplementary Tables 1, 2 and 3. The ALPS-Index is higher in HC, compared to AD in all three cohorts respectively (Table 3; Fig. 5). There was no difference in detected number nor volume of WMH between AD and HC in most cohorts, besides WMH count in DELCODE, with a higher number of WMH in AD, compared to HC (p-value = 0.001).

**Table 3:** Group differences between AD (Alzheimers diesease dementia patients) and HC (healthy controls) in the DTI_ALPS-Index

Cohort	Location	AD (mean ± SD)	HC (mean ± SD)	*P*-Value	Cohens d
ADNI	ALPS_comb	1.080 ± 0.236	1.190 **±** 0.132	0.008*	-0.575
ADNI	ALPS_R	1.088 ± 0.173	1.207 **±** 0.154	0.001*	-0.727
ADNI	ALPS_L	1.123 ± 0.186	1.174 **±** 0.147	0.149	-0.304
DELCODE	ALPS_comb	1.262 ± 0.180	1.336 **±** 0.197	0.035*	-0.392
DELCODE	ALPS_R	1.245 ± 0.208	1.332 **±** 0.254	0.048*	-0.375
DELCODE	ALPS_L	1.278 ± 0.185	1.341 **±** 0.199	0.081	-0.328
ActiGliA	ALPS_comb	1.217 ± 0.122	1.356 **±** 0.139	0.004*	-1.063
ActiGliA	ALPS_R	1.217 ± 0.183	1.336 **±** 0.165	0.055	-0.683
ActiGliA	ALPS_L	1.217 ± 0.104	1.377 **±** 0.152	0.001*	-1.229

*Abbreviation*: ALPS_R, ALPS_L: calculated ALPS-Index on right R and left L hemisphere of the brain. ALPS_comb: combined ALPS-Index, by calculating the mean of Index for right and left hemisphere. SD: Standard deviation.* significant difference with *P* < 0.05. Participants with WMH contamination of the ALPS calculation were omitted from the analysis. Calculated using an ANOVA adjusted for age and sex

### ALPS-Index in Alzheimer’s disease

In this analysis of the ALPS-Index, significant group differences between AD patients and HC are observed. In the ADNI cohort, the combined ALPS-Index (ALPS_comb) shows a significantly lower value in AD patients compared to HC (1.080 ± 0.236 vs. 1.190 ± 0.132, *p* = 0.008, Cohen’s d = -0.575). Similar findings are seen for the right hemisphere (ALPS_R) in the ADNI cohort (*p* = 0.001, Cohen’s d = -0.727).

In the DELCODE cohort, significant differences are also noted in the combined ALPS-Index (*p* = 0.035, Cohen’s d = -0.392) and the right hemisphere (*p* = 0.048, Cohen’s d = -0.375). The ActiGliA cohort demonstrates the most pronounced effect sizes, with a combined ALPS-Index of 1.217 ± 0.122 in AD patients versus 1.356 ± 0.139 in HC (*p* = 0.004, Cohen’s d = -1.063), and a particularly strong difference in the left hemisphere (*p* = 0.001, Cohen’s d = -1.229). See Table 3; Fig. 5.

### Associations between ALPS-Index and AD biomarkers

**Fig. 6:**
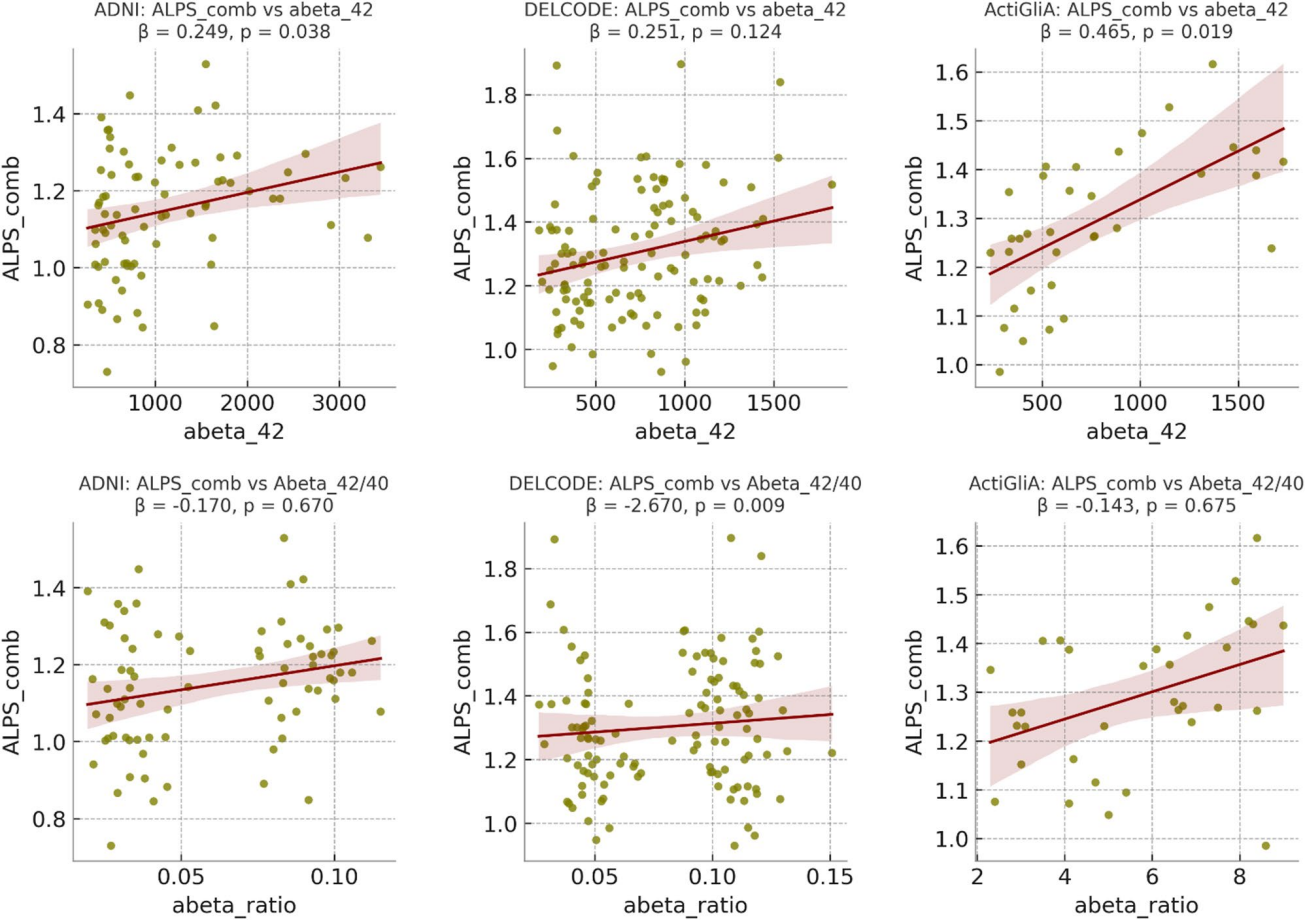
Regression plots of all 3 cohorts, outliers removed, indicating correlation between ALPS_comb and Amyloid beta values

Subsequent multivariate linear regression analyses (adjusted for age, sex, years of education and diagnose (AD, HC)) revealed correlations between the ALPS indices and Aβ42 across all three cohorts (Tables 4 and 1; Fig. 6). A significant association between Aβ ratio 42/40 and all ALPS indices was found in DELCODE (ALPS_comb: beta = -0.645; p-value = 0.009). For ActiGlia, Aβ42 showed a significant association with ALPS_L index (beta = 0.531; p-value = 0.010). For ADNI, no significant associations between ALPS indices and Aβ42 or Aβ42/40 were found (all p-values > 0.071).

**Table 4:** Correlation between ALPS indices and Amyloid-beta 42

	ActiGlia				ADNI				DELCODE			
	Aβ42				Aβ42				Aβ42			
	β	SE	Adj-*R*²	*p*-value	β	SE	Adj-*R*²	*p*-value	β	SE	Adj-*R*²	*p*-value
ALPS_comb	0.47	0.19	0.43	0.019*	0.25	0.19	0.04	0.206	0.25	0.16	0.11	0.124
ALPS_R	0.31	0.18	0.45	0.104	0.27	0.17	0.24	0.133	0.30	0.16	0.09	0.071
ALPS_L	0.53	0.19	0.39	0.010*	0.35	0.19	0.10	0.072	0.13	0.16	0.10	0.416

*Abbreviations*: ALPS_R, ALPS_L: calculated ALPS index on right R and left L hemisphere of the brain. ALPS_comb: combined ALPS index, by calculating the mean of Index for right and left hemishphere; adj-R^2^: adjusted R^2^; SE: Standard error; * significant difference with *P* < 0.05. Calculated with multivariate linear regression analyses, using age, sex years of education and diagnose (ADvsHC) as confounding variables

Additionally, we performed multivariate linear regression analyses specifically within the AD group to examine AD-specific correlations of the ALPS indices, beyond investigating group differences between HC and AD. These analyses, also adjusted for age, sex, and years of education, revealed slightly different results (see Tables 5 and 2). Amyloid-beta 42 correlates significantly with the ALPS Index on the left side of the brain in ADNI (beta = 0.43; p-value = 0.042). Where we were able to detect significant correlation in the ActiGliA cohort and Aβ42 before, we were unable to see the same results in just the AD group with p-values > = 0.249.

**Table 5:** Correlation between ALPS indices and Amyloid-beta 42 in AD

	ActiGlia				ADNI				DELCODE			
	Aβ42				Aβ42				Aβ42			
	β	SE	Adj-*R*²	*p*-value	β	SE	Adj-*R*²	*p*-value	β	SE	Adj-*R*²	*p*-value
ALPS_comb	0.18	0.00	0.39	0.471	0.09	0.00	-0.14	0.707	-0.12	0.00	0.09	0.437
ALPS_R	0.24	0.00	0.60	0.249	-0.08	0.00	-0.01	0.705	-0.06	0.00	0.13	0.679
ALPS_L	0.00	0.00	-0.12	0.997	0.43	0.00	0.24	0.042*	-0.16	0.00	0.03	0.306

*Abbreviations*: ALPS_R, ALPS_L: calculated ALPS index on right R and left L hemisphere of the brain. ALPS_comb: combined ALPS index, by calculating the mean of Index for right and left hemishphere; adj-R^2^: adjusted R^2^; SE: Standard error; * significant difference with *P* < 0.05. Calculated with multivariate linear regression analyses, using age, sex, and years of education as confounding variables

Aβ40/42-ratio correlates significantly with the combined and right ALPS-Index in DELCODE (ALPS_comb: beta = 0.255; p-value = 0.003).

## Associations between ALPS and WMH

### Subsequent analyses focused on WMHs specifically located within the 5 mm ROIs, conducted with participants who were excluded from other calculations

In the correlation analysis between ALPS indices and WMH volume, found specifically in the 5 mm-sphere ROI, the multivariate regression revealed significant associations in several cohorts. In the ADNI cohort, the right hemisphere ALPS-Index was negatively correlated with WMH volume (β = -0.217, *p* = 0.040), as was the left hemisphere ALPS-Index in the DELCODE cohort (β = -0.171, *p* = 0.049). No significant correlations were found in the ActiGlia cohort.

**Fig. 7:**
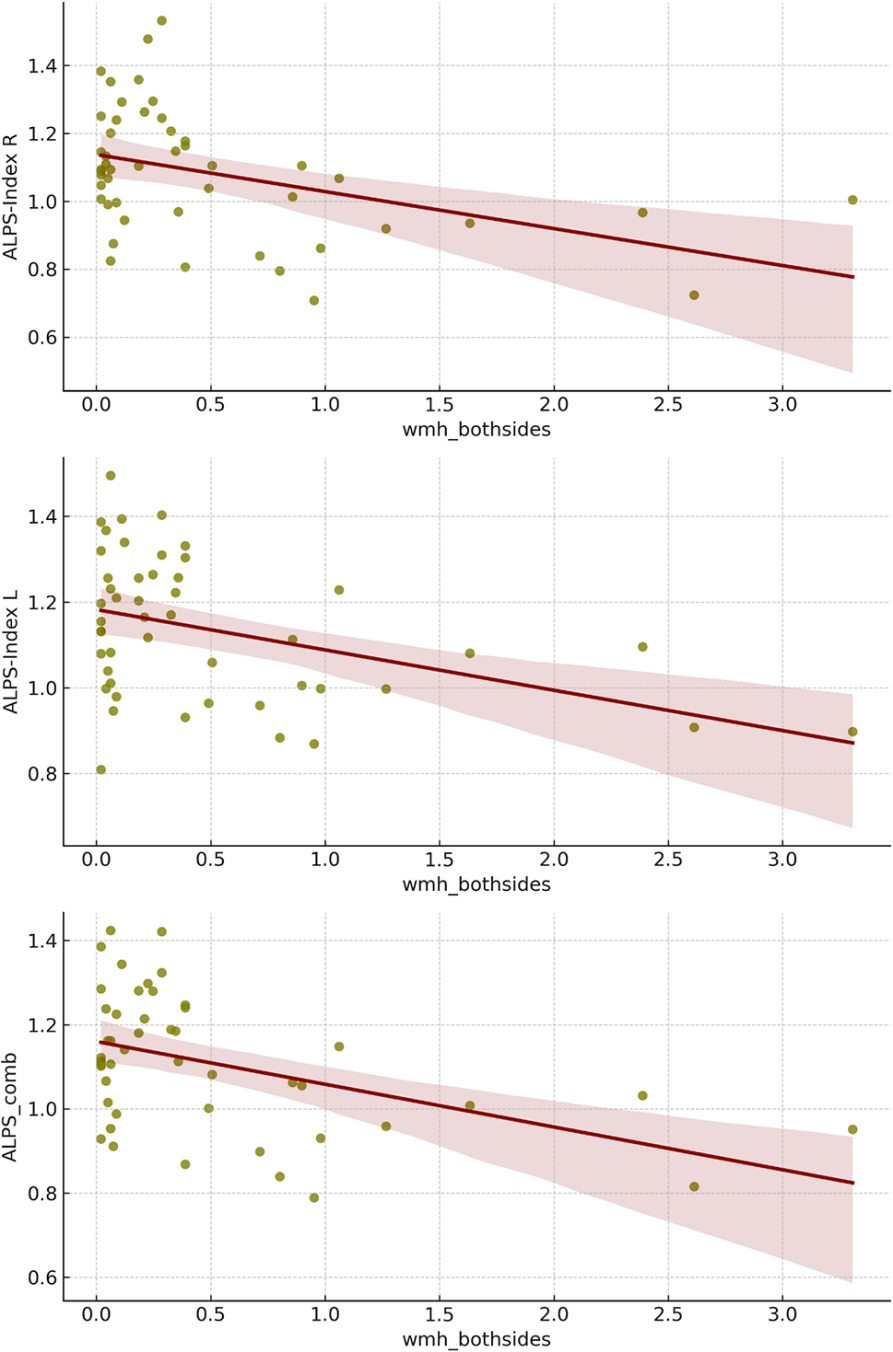
Regression plots of all 3 cohorts combined, showing volume of WMH in ROI - in the subjects excluded from the other calculations - and the different ALPS indices, indicating a negative correlation between ALPS-Index and WMH found in the specific region, where ALPS is calculated

When combining all three cohorts, a significant negative correlation was observed between WMH volume and ALPS_comb (β = 0.154, *p* = 0.010 for WMH_comb), with consistent findings for ALPS_R (β = -0.202, *p* = 0.001). See Tables 6 and 7; Fig. 7.

**Table 6:** Correlation between ALPS indices and WMH found in the 5 mm-sphere- ROI in the 3 cohorts

	WMH Volume detected in ROI (left and right side combined)					
	ActiGlia (*n* = 2)		ADNI (*n* = 36)		DELCODE (*n* = 10)	
	β	*p*-value	β	*p*-value	β	*p*-value
ALPS_comb	0.067	0.675	-0.103	0.373	-0.215	0.014*
ALPS_R	0.072	0.625	-0.217	0.040*	-0.208	0.022*
ALPS_L	0.047	0.784	-0.068	0.562	-0.171	0.049*

*Abbreviations*: ALPS_R, ALPS_L: calculated ALPS-Index on right R and left L hemisphere of the brain. ALPS_comb: combined ALPS-Index, by calculating the mean of Index for right and left hemishphere. * significant difference with *P* < 0.05. Calculated with multivariate linear regression analyses, using age, sex, diagnose and years of education as confounding variable

**Table 7:** Correlation between ALPS indices and WMH found in the 5 mm-sphere- ROI with the 3 cohorts combined (*n* = 48)

WMH Volume detected in ROI												
	WMH_comb				WMH_R				WMH_L			
	β	SE	Adj-R ²	p-value	β	SE	Adj-R ²	p-value	β	SE	Adj-R ²	p-value
ALPS_comb	0.154	0.053	0.18	0.010*	-0.142	0.084	0.18	0.018*	-0.133	0.109	0.18	0.027*
ALPS_R	-0.202	0.058	0.16	0.001*	-0.202	0.093	0.16	0.001*	-0.154	0.120	0.14	0.013*
ALPS_L	-0.117	0.051	0.17	0.055*	-0.095	0.081	0.16	0.120	-0.118	0.104	0.17	0.052

*Abbreviations: aR* ²*= adjusted R squared; SE: Standard Error;* ALPS_R, ALPS_L: calculated ALPS-Index on right R and left L hemisphere of the brain. ALPS_comb: combined ALPS-Index, WMH_R, WMH_L: volume of WMH in 5 mm sphere ROI on right and left hemisphere, WMH_comb: left and right side combined. * significant difference with *P* < 0.05. Calculated with multivariate linear regression analyses, using age, sex, diagnose and cohort as confounding variable

### Analyses focused on WMHs located in the rest of the brain, outside the 5 mm ROI, conducted with the regular participant cohort

**Fig. 8:**
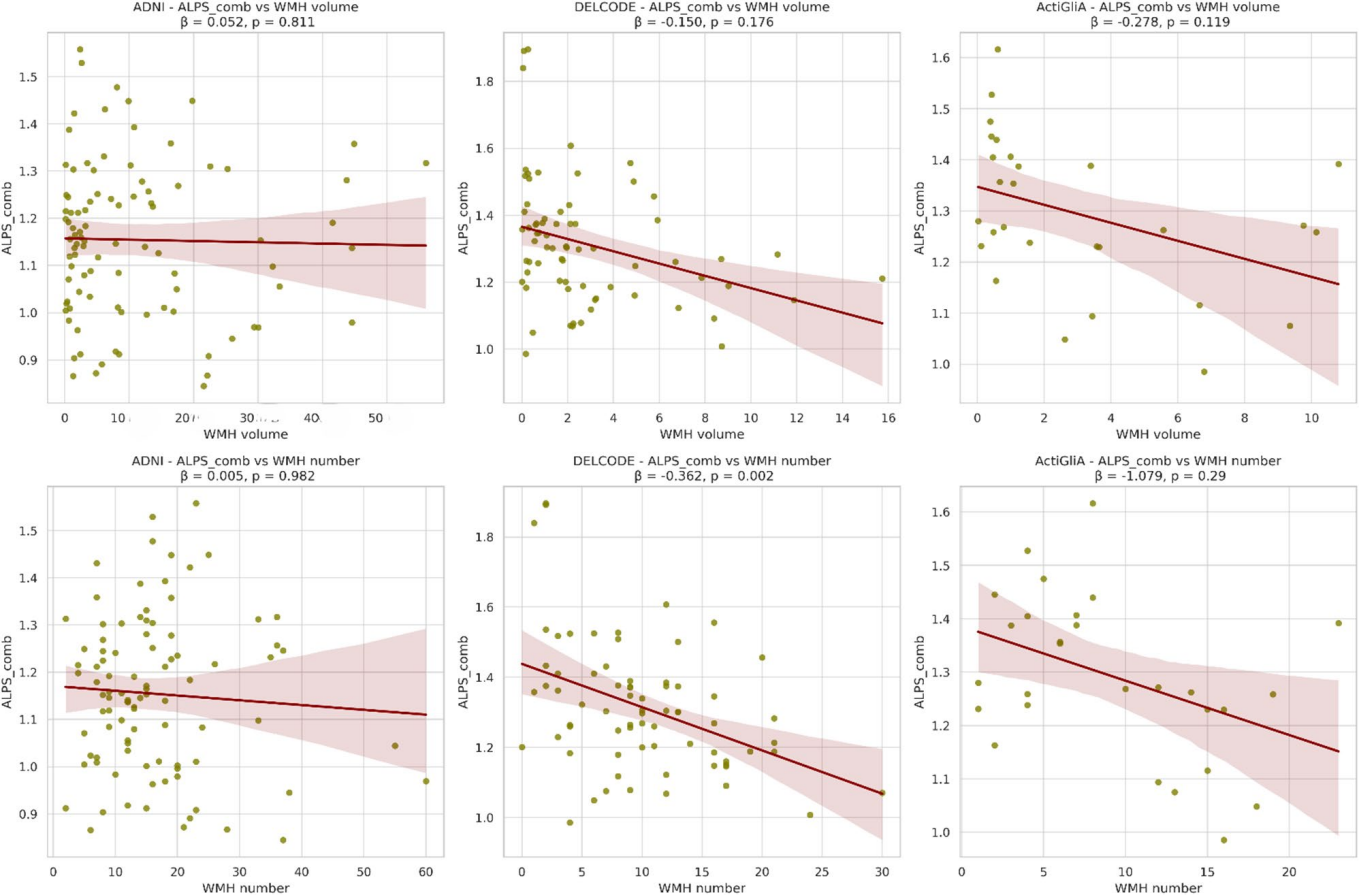
Regression plots of all 3 cohorts, outliers removed, indicating correlation between ALPS_comb and WMH findings

Distinct patterns emerged concerning WMH. In particular, a significant correlation between WMH count and the ALPS index was observed in the DELCODE cohort (Analysis with AD and HC included, but diagnose as confounding variable: ALPS_comb: beta = -0.362; p-value = 0.002. Analysis in AD group, without HC: ALPS_comb: beta = -0.377; p-value = 0.013; ALPS_L: beta = -0.416; p-value = 0.008). In the ActiGliA cohort, the p-values were not statistically significant at the 0.05 level, but there was an indication of a relationship between WMH volume and the ALPS_R index (beta = -0.290; p-value = 0.077). However, no such relationship was evident in the ADNI cohort (Fig. 8).

### ALPS-Index and its associations with clinical and cognitive markers

**Fig. 9:**
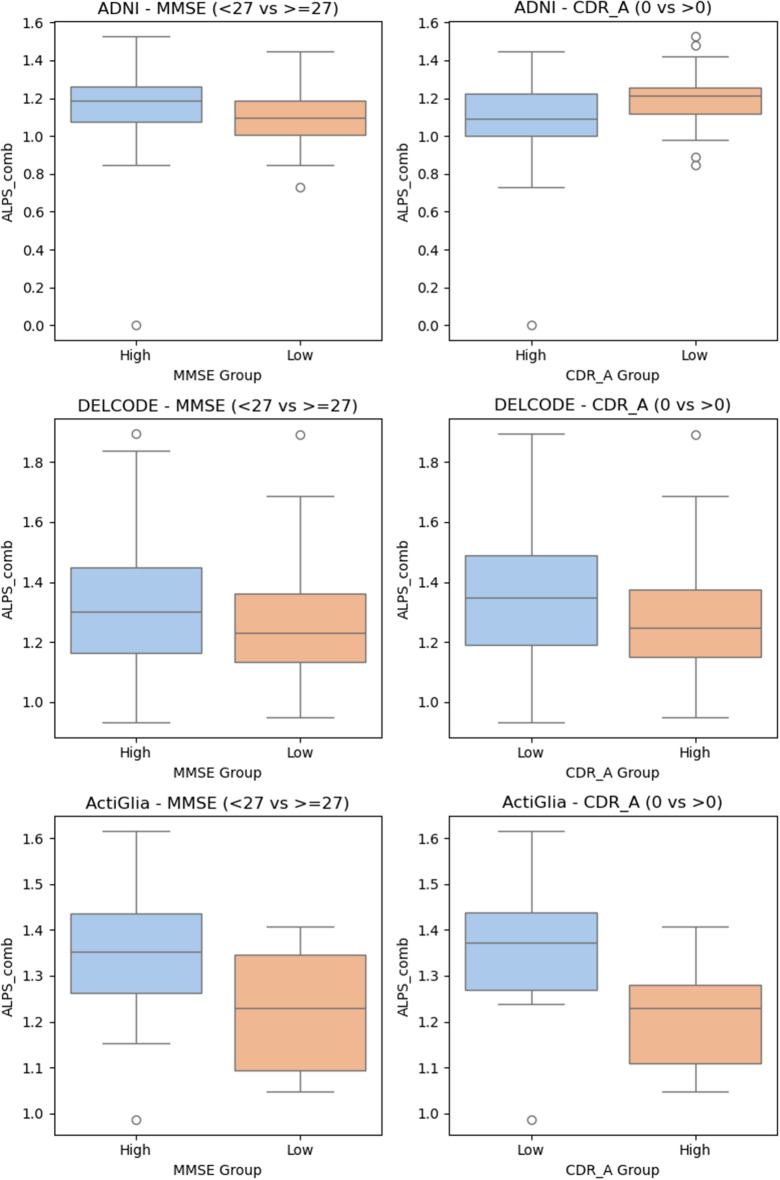
Boxplots for ALPS_comb and clinical assessment measures (MMSE: low < 27, high = > 27 or CDR: low = 0, high > 0)

In the ADNI and ActiGliA cohorts, associations were observed between the ALPS indices and cognitive assessment measures—specifically, the MMSE in ADNI (ALPS_comb: p-value: 0.038, beta:-0.307), CDR in ADNI (ALPS_L: p-value:0.035, beta:0.469) and the CDR in ActiGliA (ALPS_R: p-value:0.031, beta:-0.797) — suggesting a link with cognitive decline and disease progression. However, these correlations were not consistently replicated in the DELCODE cohort (p-value > 0.168), as seen in Fig. 9. When comparing high vs. low CDR and MMSE, the Mann-Whitney U test results show that CDR (0 vs. > 0) differences in ALPS_comb are statistically significant for all three cohorts (ADNI: *p* = 0.0082, DELCODE: *p* = 0.0244, ActiGlia: *p* = 0.0016), suggesting a meaningful association. In contrast, the MMSE (< 27 vs. ≥ 27) comparisons show weaker or no significant differences, with only ActiGliA reaching significance (*p* = 0.0072), while ADNI (*p* = 0.1285) and DELCODE (*p* = 0.0742) do not. This suggests that ALPS_comb differences are more strongly linked to CDR status than MMSE classification (Fig. 9).

## Discussion

The ALPS-Index has gained prominence as a tool for measuring the glymphatic system’s functionality in various neurodegenerative disorders, including Parkinson’s disease, frontotemporal dementia, amyotrophic lateral sclerosis, stroke, migraines, idiopathic normal pressure hydrocephalus, and traumatic brain injuries among others [39–44]. This study aimed to explore the association of the ALPS-Index with hallmarks of AD, such as cognitive decline and overall dementia severity, CSF biomarkers and markers of microvascular damage (WMH).

The main results of our study were: (i) There was a significant group differences in ALPS indices between AD patients and HC across all cohorts, with AD patients showing lower ALPS indices. (ii) ALPS-Index was positively associated with Aβ42 and cognitive and clinical decline and (iii) WMH within a ROI is leading to lower ALPS indices. Our findings confirm earlier studies that have identified the ALPS-Index as a viable marker for disease progression and potentially for early detection of AD in very early stages, mild cognitive impairment, or even before the onset of symptoms. In a study by Zhong et al., AD patients showed significantly lower ALPS-Index compared to controls, and a decreased ALPS-Index with cognitive decline [15, 45].

Furthermore, we observed strong correlations between the ALPS-Index and CSF biomarkers, particularly Aβ42 and the Aβ42/Aβ40 ratio. Higher CSF Aβ42 levels consistently correlated with higher ALPS indices across the cohorts, supporting the hypothesis that glymphatic clearance mechanisms are closely tied to amyloid-beta metabolism. Notably, this is the first study to establish such a relationship with CSF biomarkers, highlighting its unique contribution to the field of AD research. Similarly, other studies show correlations between ALPS and extracellular perivascular space (ePVS), which suggest a shared pathology between impaired glymphatic function and AD-specific markers such as amyloid-beta aggregation, as observed in both PET scans and CSF samples [28, 46, 47].

While our analysis initially showed no significant differences in WMH volume or count between AD and HC groups within the ADNI cohort, a more detailed examination within the DELCODE and ActiGlia cohort revealed significant correlations between WMH count and ALPS indices. This suggests that WMH may impact the measurement of the ALPS-Index, possibly due to changes in bulk diffusivity caused by the WMH, though an association between AD pathology and cerebrovascular factors cannot be ruled out. It has been shown in several previous studies that WMH are correlated to glymphatic impairment and the higher the vascular damage the lower the ALPS-Index [48–50]. One study in particular highlighted an interesting L-shaped association of the DTI-ALPS-Index with the presence and severity of CSVD (cortical small vessel disease) measured by taking WMH into account [50]. The graph could indicate a threshold level of glymphatic function beyond which WMH begin to significantly impact brain health. When ALPS-Index values are above this threshold, WMH values remain low and stable. However, once the ALPS-Index falls below this critical value, WMH values start to increase sharply. This suggests that a minimally functional glymphatic system might be sufficient to prevent or limit WMH, but any further decline leads to rapid deterioration. At lower levels of WMH, the vascular impairment might be mild and not significantly affect glymphatic function. However, with more extensive vascular damage (as WMH increases), there may be a critical point where glymphatic impairment becomes pronounced, leading to the observed decline in the ALPS-Index. This L-shaped association might be a reason to why especially smaller cohorts might not show too big of a correlation between ALPS and WMH. Another study was able to show how DTI-ALPS-Index partially mediated the association of Choroid plexus volume (CPv) with both WMH load and growth and how CPv was correlated with slower glymphatic clearance in the brain– again highlighting the close relationship between WMH, vascular health and the clearance of the brain done by the GS [49].

Specifically, there was a negative correlation between WMH in the ROI and ALPS, likely due to vascular impairments in these regions affecting measurements of diffusivity along the x-axis. These findings were significant in both the ADNI and DELCODE cohorts but not in ActiGliA, likely due to the small number of subjects with WMH in these specific ROIs (*n* = 4). Once we combined all three cohorts together and looked at correlation with age, sex, and group as confounding variables, we found significant negative correlations in all three ALPS indices, likely due to vascular impairments in these regions affecting measurements of diffusivity along the x-axis. This highlights the necessity of considering vascular damage when interpreting ALPS-Index changes, as such damage can mimic or obscure changes attributable to AD pathology.

Crucially, our findings also revealed significant correlations between MMSE and CDR scores and the ALPS-Index across all three cohorts, indicating that glymphatic system impairment is associated with decline in cognitive performance and increased dementia severity. These findings are in line with several previous studies suggesting a correlation between ALPS-Index and cognitive decline in AD [11, 15, 27].

The observed differences in ALPS indices between the left and right hemispheres suggest variations in diffusivity along perivascular spaces, potentially reflecting asymmetries in glymphatic system function. Recent studies have revealed asymmetry in the glymphatic system function between brain hemispheres, with observations of a leftward asymmetry in healthy adults. It is hypothesized that the glymphatic system may function as a separate system in the left and right hemispheres [51]. Several factors could contribute to these hemispheric differences. Anatomical variations in the vascular and perivascular architecture between hemispheres may influence the efficiency of glymphatic clearance [52]. Functional lateralization of the brain, particularly differences in neuronal activity or metabolic demands, could also play a role, as glymphatic function is closely tied to cerebrovascular dynamics and interstitial fluid flow. Furthermore, asymmetrical distribution of white matter hyperintensities or regional variations in AQP4 expression, which regulates fluid transport, might differentially affect glymphatic function. These findings underscore the need for further research to understand how hemispheric differences in glymphatic activity might contribute to or result from neurodegenerative processes like Alzheimer’s disease.

While our study provides valuable insights into the importance of glymphatic impairment in AD, it is important to acknowledge several limitations that may affect the generalizability and interpretation of our findings. Potential for WMH to confound the ALPS-Index calculations remains a critical concern. The ALPS-Index is predicated on assessing changes in diffusivity along the x-axis, representing alterations in fluid dynamics within perivascular spaces—a key factor in AD pathology.

One significant concern is that the ALPS index primarily measures predominant water diffusion along the perivascular space, yet it does not directly validate glymphatic system functionality. Moreover, this method lacks the ability to differentiate between water movement within or outside the perivascular space and cannot assess the direction of flow. This measurement can be confounded by the presence of vascular damage, such as that represented by WMH, which can similarly affect diffusivity metrics. Further research is essential to develop strategies that effectively differentiate between changes due to perivascular space alterations and those resulting from vascular damage. Additionally, variations in imaging techniques, resolution, and ROI placement impact the reproducibility and interpretation of the ALPS index. To our knowledge, this is the first study that incorporates WMH into the calculation of the ALPS-Index [11, 27, 53]. This study’s exclusion of individuals with pronounced WMH within the ROI was an attempt to address this issue; however, future studies should incorporate more comprehensive methodologies to mitigate such confounding factors. Our study’s scope was limited to early-stage AD cases, primarily within demographically homogeneous populations. Future studies should aim to include more diverse populations to enhance the representativeness and generalizability of the findings, and they should incorporate follow-up periods to assess the enduring effects of the variables studied. We were unable to control for all potential confounding variables, such as lifestyle factors and genetic predispositions, which might influence the results. Future studies should consider a broader range of confounding factors. The relatively small sample size for WMH in ROI may limit the statistical power to detect significant differences and relationships.

Lastly, other studies also touched upon the role of inflammation, microglial activation, and reactive astrogliosis in impairing aquaporin-4 (AQP4), leading to discrepancies in CSF flow and accumulation of brain waste products such as Aβ and tau [31, 54–56]. Extending this speculation, perhaps regional loss of AQP4 may explain subregion-dependent susceptibility to neurodegeneration by driving local interstitial fluid and protein stagnation increasing the risk of aggregation prone proteins. This inflammation might be a precursor to or a consequence of protein aggregation, suggesting a complex, possibly bidirectional relationship between neuroinflammation and neurodegeneration. The interplay between AQP4, neuroinflammation, and the glymphatic system may contribute to the progression of neurodegenerative processes [57].

While there are some similarities between our study and the work by Shu-Yi Huang et al., 2024 [46], there are several significant differences that distinguish our research. The previous study primarily focuses on longitudinal cohorts and examines how ALPS might be used as a clinical marker to track disease progression or detect Alzheimer’s disease. In contrast, our study utilizes three distinct cohorts in a cross-sectional analysis to explore how ALPS correlates with WMH and other AD-specific biomarkers, with a particular focus on WMH found in the ROI.

One of the key distinctions in our methodology is the use of WMH as a biomarker, specifically excluding participants with WMH in the ROI, allowing us to investigate the localized effects of WMH on ALPS. This aspect was not specifically addressed in the earlier study. Additionally, while the previous study utilized the PACC to measure cognitive function, we used MMSE and CDR, which are more commonly used in clinical settings and provide different insights into cognitive status in our cohort.

Overall, our study aims to provide new insights into how WMH in the ROI influences ALPS and its potential to correlate with other Alzheimer’s disease biomarkers, offering a novel approach distinct from the longitudinal focus of the prior research.

In conclusion, our findings underscore the potential of the ALPS-Index as a biomarker for Alzheimer’s Disease, albeit with considerations for the influence of WMH on its accuracy and reliability. The variations observed across cohorts highlight the complexity of AD pathology and the need for comprehensive approaches in biomarker development, including associative analyses beyond imaging, such as those offered by animal models, histological examinations, and other investigative methods. Further studies are needed to elucidate the role of cerebrovascular factors in AD and refine neuroimaging biomarkers for early diagnosis and disease monitoring, with a particular focus on cognitive implications in our aging population.

## Electronic supplementary material

Below is the link to the electronic supplementary material.

Supplementary Material 1

## Data Availability

No datasets were generated or analysed during the current study.

## Declarations

### DELCODE study group

The DELCODE Study Group comprises investigators from multiple sites who have contributed to the design, data acquisition, or analysis of the DELCODE study. Specific authors listed in the main author list (Julian Hellmann-Regen^10,11,12^, Josef Priller^10,14,15,16^, Anja Schneider^17,18^, Frank Jessen^17,19,20^, Emrah Düzel^21,22^, Katharina Buerger^4,23^, Stefan Teipel^24,25^, Christoph Laske^26,27^, Oliver Peters^10,13^, Eike Spruth^10,14^, Klaus Fliessbach^17,18^, Ayda Rostamzadeh^19^, Wenzel Glanz^21^, Daniel Janowitz^23^, Ingo Kilimann^24,25^, Sebastian Sodenkamp^26,28^, Michael Ewers^4,23^) are also part of the DELCODE consortium.

### Alzheimer’s disease neuroimaging initiative (ADNI)

Data used in preparation of this article were obtained from the Alzheimer’s Disease Neuroimaging Initiative (ADNI) database (adni.loni.usc.edu). As such, the investigators within the ADNI contributed to the design and implementation of ADNI and/or provided data but did not participate in analysis or writing of this report. A complete listing of ADNI investigators can be found at: http://adni.loni.usc.edu/wp-content/uploads/how_to_apply/ADNI_Acknowledgement_List.pdf.

## Abbreviations

AD: Alzheimer‘s disease
Aβ: amyloid beta
DTI-ALPS: Diffusion tensor Imaging along perivascular spaces
HC: healthy controls
CVD: cerebrovascular disease
ADS: Alzheimer’s disease subjects
WMH: White matter hyperintensities
PVS: Perivascular space
FLAIR: fluid attenuated inversion recovery
LST: lesion segmentation tool
ROI: Region of interest
CSF: cerebrospinal fluid
MMSE: Mini Mental state examination
CDR: clinical dementia rating
BBB: Blood brain barrier
ISF: interstitial fluid
FA: fractional anisotropy
SD: standard deviation
ALPS_comb: left and right ALPS-Index combined
ALPS_R: right ALPS-Index
ALPS_L: left ALPS-Index
ePVS: enlarged perivascular space
CSVD: cortical small vessel disease
CPv: choroid plexus volume
AQP4: Aquaporin 4 channel
M/F: male/female
TR: repetition time
TE: echo time
FoV: field of view
