# Supplementary material for: Perivascular space and white matter hyperintensities in Alzheimer’s disease: associations with disease progression and cognitive function
Source: Alzheimers Res Ther. 2025 Mar 18;17:62. doi: 10.1186/s13195-025-01707-9 (PMC11917016; doi:10.1186/s13195-025-01707-9)
Supplement: Supplementary file 1 — Supplementary Material 1 [file 13195_2025_1707_MOESM1_ESM.docx]

**Supplementary material:**

**ADNI**

| **Supplementary Table 1**  *Sociodemographic and biomarker differences in the ADNI cohort* | | | |  |
| --- | --- | --- | --- | --- |
|  | **HC(n=49)** | **AD(n=43)** | **p-value** | **Cohens d** |
|  |  |  |  |  |
| age(years), SD | 69.8 ± 6.8 | 73.7 ± 7.6 | .013* | 0.54 |
| sex(M/F) | 12/24 | 17/13 | .031* |  |
| yearsofeducation, SD | 16.6 ± 2.6 | 15.9 ± 4 | .367 | 0.21 |
| MMSE, SD | 29.2 ± 0.9 | 25.2 ± 4.3 | .000* | 1.33 |
| CDR, SD | 0 ± 0 | 0.7 ± 0.4 | .000* |  |
| Aβ42 (pg/ml), SD | 1618.7 ± 765.8 | 923.7 ± 1650 | .021* | 0.55 |
| Aβ42_40_ratio, SD | 0.09 ± 0.013 | 0.03 ± 0.007 | .000* | 5.66 |
| WMH_number, SD | 12.73 ± 7.39 | 15.55 ± 9.01 | .361 | 8.16 |
| WMH_volume (ml), SD | 6.164 ± 10.631 | 7.141 ± 7.992 | .665 | 0.58 |
| ALPS_comb, SD | 1.19 ± 0.13 | 1.08 ± 0.24 | .008* | 0.58 |
| ALPS_R, SD | 1.21 ± 0.15 | 1.09 ± 0.17 | .001* | 0.75 |
| ALPS_L, SD | 1.17 ± 0.15 | 1.12 ± 0.19 | .149 | 0.29 |
| *Abbreviations:* M/F, male/female; HC, healthy controls; AD, Alzheimer's Disease; MMSE. Mini-Mental State Examination; ALPS, along perivascular spaces; comb, Left and Right Index combined, L, left; R, right; WMH, White Matter hyperintensities; missing sex (n=26) * significant difference with P < 0.05, in a two-sided, two sample T-test. | | | | |

**ActiGliA**

| **Supplementary Table 2**  *Sociodemographic and biomarker differences in the ActiGliA cohort* | | | |  |  |
| --- | --- | --- | --- | --- | --- |
|  | | | |  |  |
|  | **HC(n=18)** | **AD(n=16)** | **p-value** | **Cohens d** |  |
|  |  |  |  |  |  |
| age(years), SD | 70.94 ± 7.08 | 70.75 ± 7.47 | .938 | 0.03 |  |
| sex(M/F) | 9/9 | 10/6 | .479 |  |  |
| yearsofeducation, SD | 15.19 ± 3.87 | 15.31 ± 4.87 | .938 | -0.03 |  |
| MMSE, SD | 29.56 ± 0.71 | 24.13 ± 2.99 | .000* | 2.57 |  |
| CDR, SD | 0 ± 0 | 0.56 ± 0.17 | .000* |  |  |
| Aβ42 (pg/ml), SD | 1022.73 ± 480.34 | 469.26 ± 143.03 | .000* | 2.08 |  |
| Aβ42_40_ratio, SD | 7.39 ± 0.96 | 3.70 ± 0.97 | .000* | 3.84 |  |
| WMH_number, SD | 7.41 ± 6.14 | 18.8 ± 6.73 | .147 | 6.43 |  |
| WMH_volume (ml), SD | 2.86 ± 3.62 | 6.38 ± 11.88 | .283 | 0.41 |  |
| ALPS_comb, SD | 1.36 ± .014 | 1.22 ± 0.10 | .004* | 1.14 |  |
| ALPS_R, SD | 1.34 ± 0.16 | 1.22 ±0.18 | .055 | 0.71 |  |
| ALPS_L, SD | 1.38 ± 0.15 | 1.22 ± 0.1 | .001* | 1.23 |  |
| *Abbreviations:* M/F, male/female; HC, healthy controls; AD, Alzheimer's Disease; MMSE. Mini-Mental State Examination; ALPS, along perivascular spaces; comb, Left and Right Index combined, L, left; R, right; WML, White Matter lesions; * significant difference with P < 0.05, in a two-sided, two sample T-test. | | | | |  |

**DELCODE**

| **Supplementary Table 3**  *Sociodemographic and biomarker differences in the Delcode cohort* | | | | |  |  | |  |
| --- | --- | --- | --- | --- | --- | --- | --- | --- |
|  | **HC(n=67)** | **AD(n=54)** | **p-value** | **Cohens d** | | |  | |
| age(years), SD | 69.4 ±6.1 | 73.4 ± 6.5 | .001* | 0.64 | | |  | |
| sex(M/F) | 34/33 | 27/27 | .936 |  | | |  | |
| yearsofeducation, SD | 14.5 ± 2.7 | 13.8 ± 2.9 | .199 | 0.25 | | |  | |
| MMSE, SD | 29.4 ± 0.9 | 26 ± 3.1 | .000* | 1.57 | | |  | |
| CDR, SD | 0 ± 0 | 0.58 ± 0.19 | .000* |  | | |  | |
| Aβ42 (pg/ml), SD | 994.22 ± 248.89 | 383.39 ± 110.2 | .000* | 3.07 | | |  | |
| Aβ42_40_ratio, SD | 0.108 ± 0.013 | 0.047 ± 0.01 | .000* | 5.08 | | |  | |
| WMH_number, SD | 6.67 ± 4.55 | 13.07 ± 9.32 | .001* | 0.9 | | |  | |
| WMH_volume (ml), SD | 2.31 ± 6.25 | 5.31 ± 8.03 | .093 | 0.42 | | |  | |
| ALPS_comb, SD | 1.34 ± 0.197 | 1.26 ± 0.18 | .035* | 0.42 | | |  | |
| ALPS_R, SD | 1.33 ± 0.254 | 1.25 ± 0.185 | .048* | 0.35 | | |  | |
| ALPS_L, SD | 1.34 ± 0.199 | 1.28 ± 0.185 | .081 | 0.31 | | |  | |
| *Abbreviations:* M/F, male/female; HC, healthy controls; AD, Alzheimer's Disease; MMSE. Mini-Mental State Examination; ALPS, along perivascular spaces; comb, Left and Right Index combined, L, left; R, right; WML, White Matter lesions; * significant difference with P < 0.05, in a two-sided, two sample T-test. | | | | | | |  | |
